# Ultrahigh photoconductivity of bandgap-graded CdS_*x*_Se_*1*−*x*_ nanowires probed by terahertz spectroscopy

**DOI:** 10.1038/srep27387

**Published:** 2016-06-06

**Authors:** Hongwei Liu, Junpeng Lu, Zongyin Yang, Jinghua Teng, Lin Ke, Xinhai Zhang, Limin Tong, Chorng Haur Sow

**Affiliations:** 1 Institute of Materials Research and Engineering, Agency for Science, Technology and Research (A*STAR), 2 Fusionopolis Way, Innovis, #08-03, 138634, Singapore; 2 Department of Physics, 2 Science Drive 3, National University of Singapore, 117542, Singapore; 3 State Key Laboratory of Modern Optical Instrumentation, Department of Optical Engineering, Zhejiang University, Hangzhou, Zhejiang, 310027, China; 4 Department of Electrical and Electronic Engineering, South University of Science and Technology of China, 1088 Xueyuan Road, Nanshan District, Shenzhen, Guangdong, 518055, China

## Abstract

Superiorly high photoconductivity is desirable in optoelectronic materials and devices for information transmission and processing. Achieving high photoconductivity *via* bandgap engineering in a bandgap-graded semiconductor nanowire has been proposed as a potential strategy. In this work, we report the ultrahigh photoconductivity of bandgap-graded CdS_*x*_Se_*1*−*x*_ nanowires and its detailed analysis by means of ultrafast optical-pump terahertz-probe (OPTP) spectroscopy. The recombination rates and carrier mobility are quantitatively obtained *via* investigation of the transient carrier dynamics in the nanowires. By analysis of the terahertz (THz) spectra, we obtain an insight into the bandgap gradient and band alignment to carrier transport along the nanowires. The demonstration of the ultrahigh photoconductivity makes bandgap-graded CdS_*x*_Se_*1*−*x*_ nanowires a promising candidate as building blocks for nanoscale electronic and photonic devices.

II–VI semiconductor nanowires have arisen as versatile building blocks for compact devices and components in nanoelectronics and nanophotonics. Exploiting the unique one-dimensional architecture, high aspect ratio and large surface-to-volume ratio, semiconductor nanowires have been designed or assembled into various electronic or photonic circuits as waveguides, lasers, field-effect transistors, photodetectors and light-emitting diodes^1^,^2^,^3^,^4^,^5^,^6^,^7^,^8^. Bandgap is an intrinsic character of a semiconductor and dictates many of its properties, for example, the light absorption and emission of a semiconductor and the operating wavelength of an optoelectronic device. To optimize the performance of optoelectronic devices, bandgap engineering is invoked as one of effective techniques to alter the physical properties of semiconductors, besides methods of selective area growth and quantum well intermixing^9^,^10^,^11^. One of the effective bandgap engineering techniques for II–VI semiconductor nanowires is to tune the constituent composition of alloys during the growth or assembly of semiconductor nanowires. Ternary or quaternary alloyed semiconductor nanowires of ZnSSe, CdSSe, ZnCdSSe *etc*. have been developed^12^,^13^,^14^. These alloyed nanowires possess wide-tunable bandgaps covering the operating spectrum range from ultraviolet to near infrared, which provide a new platform for multicolored display and lighting, tunable wavelength lasers, multispectral photodetectors and full spectrum solar cells^14^,^15^,^16^,^17^. Nevertheless, the practical applications of the existing as-synthesized alloy nanowires are restricted by their relatively low photoconductivity. Recently, Gu *et al*. demonstrated the bandgap engineering along an individual nanowire by the synthesis of bandgap-graded CdS_*x*_Se_*1*−*x*_ nanowires^18^. Wavelength tunable photoluminescence (PL) along the nanowire has been observed. Nanophotonic devices such as color selective and controllable lasers and waveguides have been demonstrated using these nanowires^16^,^19^. Due to the bandgap gradient and “type II” band alignment along the nanowire, high photoconductivity is expected from these bandgap-graded nanowires. Herein, we employ optical-pump terahertz-probe (OPTP) spectroscopy to characterize the photoconductivity of the bandgap-graded CdS_*x*_Se_*1*−*x*_ nanowires. Ultrahigh transient photoconductivity up to 2000 Ω^−1^cm^−1^ with the free carrier density up to 3 × 10^19^ cm^−3^ is measured. We also investigate the ultrafast carrier dynamics to show a clear picture on the bandgap engineering along the composition grading and reveal the effect of the build in “type II” alignment.

## Materials and Methods

The bandgap-graded CdS_*x*_Se_*1*−*x*_ nanowires were grown on sapphire substrate by a source-moving chemical vapor deposition technique, which employed a specially designed horizontal tube furnace. The detailed growth processes were described in previous report^18^. The carrier dynamics and photoconductivity were investigated using OPTP spectroscopy. The fundamental output from a Ti:sapphire regenerative amplifier laser system (Coherent Legend), which provided ~35 fs laser pulse centered at 800 nm and at 1 kHz repetition rate, was divided into 3 paths: (1) the pump to photoexcite the sample. The 400 nm wavelength excitation laser pulses were generated by a *β*-barium borate (BBO) crystal *via* frequency doubling of the fundamental output. Optical pump fluences varied from 4 to 40 μJ/cm^2^. (2) to generate THz wave *via* air-plasma technique with frequency range from 1 to 5 THz and (3) a gate pulse for THz air-biased-coherent-detection (THz-ABCD). There were two modes of operation for OPTP measurements: pump scan and probe scan. In a pump scan, delay stage in THz-generation path was fixed and delay stage in optical pump path was moved thus THz waveform was being sampled and the changes of the THz peak transmission were recorded. In a probe scan, delay stage in optical pump path kept fixed and the delay line in THz-generation path was moved. At this time THz pulse was recorded. All experiments were conducted in N_2_ environment.

PL spectra from selected positions along the length of a bandgap-graded wire were measured by spatially resolved micro-PL. The output of a 405 nm laser was focused onto nanowires using ×50 microscope objectives with numerical aperture of 0.42, which was also used for the collection of the PL signals from the nanowires. The laser spot size was less than 2 μm and the typical length of nanowires was 100–200 μm. We scanned the focused laser spot along the nanowires and get the PL from different positions.

## Results and Discussion

**Figure 1:**
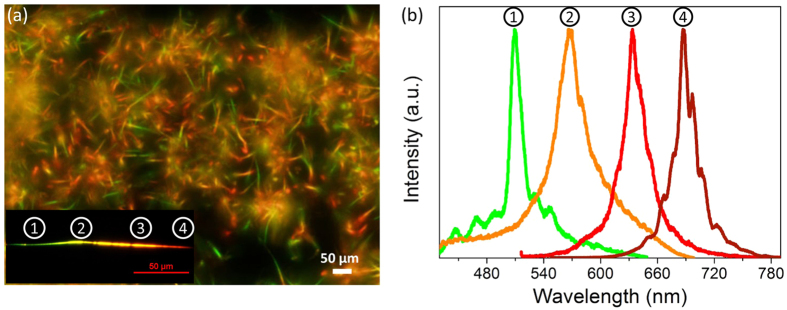
(**a**) Fluorescence microscopy image of the as-grown bandgap-graded CdS_*x*_Se_*1*−*x*_ nanowires. Insert: Fluorescence microscopy image of an individual bandgap-graded CdS_*x*_Se_*1*−*x*_ nanowires. (**b**) PL spectra collected along different positions in the individual nanowire. The intensity is normalized.

The fluorescence property of the bandgap-graded nanowires was revealed by the microscopy image as illustrated in Fig. 1a. The as-grown nanowires were excited by 405 nm laser. As shown by the captured image, all the nanowires show multicolor photoluminescence, which varied from green (CdS end) to red (CdSe end) along their length. The continuous color variation indicates the gradual variation of band gap along the length of the nanowires. PL spectra taken at selective positions along an individual nanowire are shown in Fig. 1b. Remarkably, different sites exhibits different PL emission bands with peak position gradually moving from 510 nm to 690 nm, covering most of the visible spectrum range.

**Figure 2:**
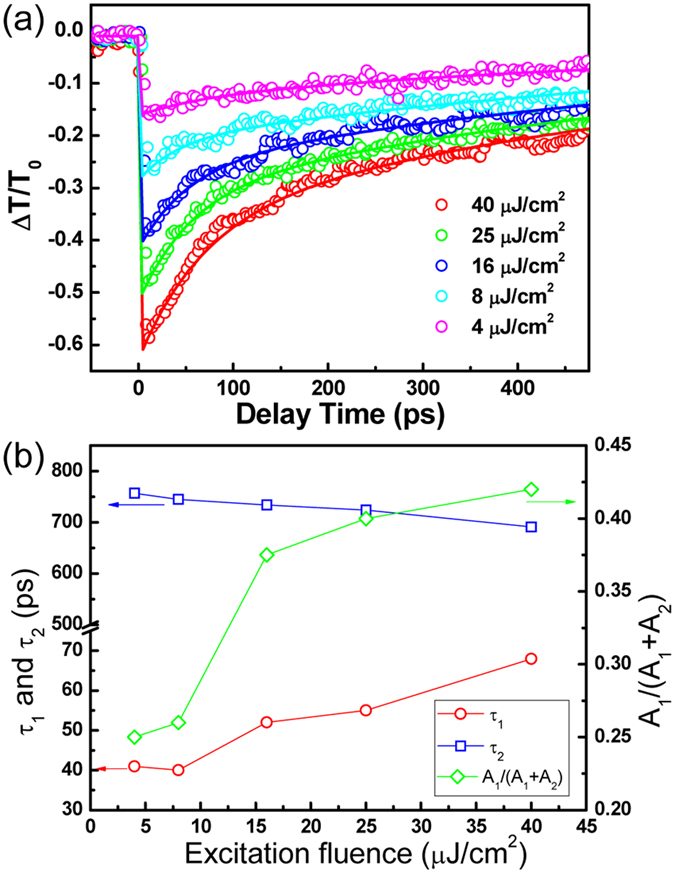
(**a**) Time-dependent differential THz transmission of representative graded CdS_*x*_Se_*1*−*x*_ nanowires at different excitation fluences. All transients are well fitted by a biexponential function. (**b**) Extracted parameters from the fitted biexponential function: *τ*_*1*_ (red circles), *τ*_*2*_ (blue squares) and *A*_*1*_/(*A*_*1*_ + *A*_*2*_) (green diamonds) plotted as a function of excitation fluence.

The dynamics of photogenerated carriers were investigated by OPTP spectroscopy. The transient THz peaks were recorded by measuring the pump-induced changes of THz peak signals as a function of the pump-probe delay time. Figure 2a illustrates the differential transmission signal Δ*T*/*T*_0_ of bandgap-graded CdS_*x*_Se_*1*−*x*_ nanowires as a function of delay time *t*. Here Δ*T* is the time-dependent transmission change of THz wave due to optical excitation, and *T*_0_ is the transmitted intensity of THz wave without optical excitation. The nanowires were excited with a series of excitation fluence of 40, 25, 16, 8 and 4 μJ/cm^2^. Evidently, the amplitude of Δ*T*/*T*_0_ drops instantaneously, following a biexponential trend after optical pumping. The experimental curves are well fitted by a biexponential function, 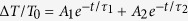, where *A*_*i*_ is the weighting factor and *τ*_*i*_ is the decay time constant. The decay time constant characterizes the average time it takes for a carrier to recombine, which indicates how fast the carriers recombine to its ground state by *i*th path. The best fitted parameters at different excitation fluences are plotted in Fig. 2b. The two processes can be assigned to surface trapping and structural-defect-related near band-edge recombination, respectively, according to our previous studies^20^,^21^. The two processes are consistent with those of uniform CdS_*x*_Se_*1*−*x*_ nanowires which have a single bandgap. The comparison of the single bandgap CdS_*x*_Se_*1*−*x*_ and bandgap-graded CdS_*x*_Se_*1*−*x*_ nanowires is depicted in Supplementary Information S1. Obviously, the characteristic decay times *τ*_*1*_and *τ*_*2*_ of the photocarriers are faster while *A*_*1*_/(*A*_*1*_ + *A*_*2*_) is smaller in bandgap-graded CdS_*x*_Se_*1*−*x*_ nanowires, indicating the impact of the surface effects in the graded nanowires is restrained. The photocarrier decay is dominated by the structural-defect-related recombination. In single-bandgap CdS_*x*_Se_*1*−*x*_ nanowires, the structural defects mainly come from the composition disorder in ternary compound, which results in statistically distributed fluctuations of an average potential. In addition to the composition disorder, the Se substitution rate in the composition graded CdS_*x*_Se_*1*−*x*_ nanowires varies along the nanowire length direction. This facilitates a stepwise structure of the nanowire band edge and promotes the formation of interface defects between different compositions along the nanowires. Therefore, in bandgap-graded CdS_*x*_Se_*1*−*x*_ nanowires, the high structural defect density leads to faster recombination, *i.e.* shorter *τ*_*2*_ of graded sample (691 ps) than composition uniform samples (>1000 ps), as depicted in Table S1b. It should be mentioned that the structural defects in bandgap-graded CdS_*x*_Se_*1*−*x*_ nanowires can impede free carriers from migrating to surface through scattering or carrier localization. Thus higher structural defect density in graded CdS_*x*_Se_*1*−*x*_ nanowires results in less carriers migrating to the surface, which reduce the possibility of carrier trapping to surface defects.

**Figure 3:**
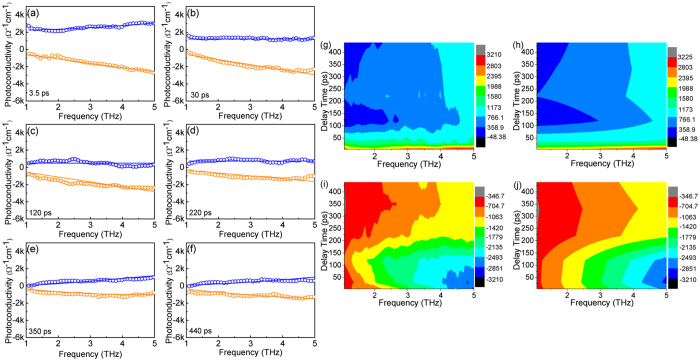
Real (blue circles) and imaginary (orange circles) part of complex photoconductivity of graded CdS_*x*_Se_*1*−*x*_ nanowires recorded at (**a**) *t* = 3.5, (**b**) 30, (**c**) 120, (**d**) 220, (**e**) 350 and (**f**) 440 ps. The solid lines are fitted by Drude-Smith model. Contour mapping of experimental ((**g**) real part, (**i**) imaginary part) and fitting ((**h**) real part, (**j**) imaginary part) results of frequency-dependent photoconductivity as a function of the delay time.

**Figure 4:**
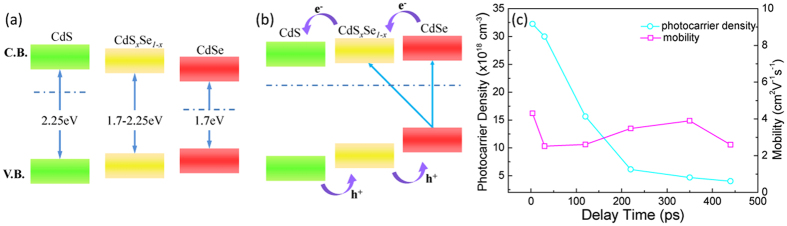
Schematic band diagrams for CdS, CdS_*x*_Se_*1*−*x*_, CdSe (**a**) before and (**b**) after the Fermi level alignment. Dash lines indicate the Fermi level before and after the Fermi level alignment. (**c**) Photocarrier density and electron mobility of representative graded CdS_*x*_Se_*1*−*x*_ nanowires plotted as a function of delay time.

Information provided by non-equilibrium state of the ternary compound under photon excitation is crucial for optimization of nanoscale optoelectronic devices. To extract this information, the pump-probe delay was fixed at a certain position and then the probe scans were collected to extract photoconductivity. From the transmitted THz electric field *E*(*t*) and the photoinduced change in THz electric field Δ*E*(*t*), we extract the complex photoinduced change in the complex optical conductivity Δ*σ*(*ω, t*) (referred to as photoconductivity throughout this paper) as a function of *t*. As a result, the photoinduced conductivity can be extracted from the recorded transient changes in THz transmission. A typical transient change in THz electric field is shown in Fig. S2. The photoconductivity can be derived from^20^,^22^


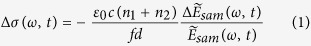


where *d* is the thickness of the sample, *ε*_*0*_ is vacuum permittivity, *c* is speed of light, *n*_1_ and *n*_2_ are the refractive indices of ambient (N_2_) and substrate (sapphire), *f* is filling factor, *t* is the pump-probe delay time, and 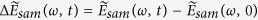, where 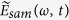 and 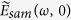 are the transient field of THz probe through the sample with and without photoexcitation. Figure 3a–f show the photoconductivity of bandgap-graded CdS_*x*_Se_*1*−*x*_ nanowires recorded at different delay times after photoexcitation. Figure 3a presents the photoconductivity at *t* = 3.5 ps, when the nanowires are totally excited and Δ*T*/*T*_0_ shows the maximum amplitude. With increasing delay time, the free carriers start to recombine and the corresponding conductivities were recorded at *t* = 30, 120, 220, 350 and 440 ps, as presented in Fig. 3b–f, respectively. More intuitive observation is displayed by the contour map shown in Fig. 3g–j, which illustrates experimental and fitted complex photoconductivity as a function of pump-probe delay time. The measured real part of photoconductivity (blue circles) can be as high as 2000 Ω^−1^cm^−1^, which is much higher than the photoconductivity of the composition uniform CdS_*x*_Se_*1*−*x*_ nanowires (900 Ω^−1^cm^−1^)^20^, other II–VI compound nanostructures (ZnO, 20 Ω^−1^cm^−1^, 10K) and III–V compound nanowires (GaAs, 10 Ω^−1^cm^−1^; GaN, 0.5 Ω^−1^cm^−1^)^23^,^24^,^25^,^26^. To the best of our knowledge, this is the highest photoconductivity value in semiconductor 1D nanostructures measured *via* similar THz characterization techniques. The high photoconductivity in graded CdS_*x*_Se_*1*−*x*_ nanowires is directly correlated with the band alignment in the nanowires, as displayed in Fig. 4a,b. As described and discussed in previous reports^27^,^28^, the band structure of individual CdS and CdSe can be schematically illustrated in Fig. 4a. After the creation of composition gradient, the Fermi level at the CdS/CdS_*x*_Se_*1*−*x*_/CdSe interface is aligned. The band edges shift upward and downward for CdSe (Se-rich component) and CdS (S-rich component) respectively, and hence the conduction band and the valence band of CdS (S-rich component) locate below corresponding edges of CdSe (Se-rich component). Consequently, the band edge of the nanowires forms a stepwise structure, producing a “type-II” band structure, as shown in Fig. 4b. Such “type-II” formation in different nanostructures, such as Zn_3_P_2_/ZnO Core/Shell Nanowires and ZnO/ZnSe Core/Shell Nanowire, has also been observed by other groups before^29^,^30^,^31^. Under photoexcitation, CdS, CdS_*x*_Se_*1*−*x*_, and CdSe absorb photons corresponding to their bandgaps (from 1.7–2.25 eV). Then the produced photoelectrons are driven to the lower conduction band of the CdS (S-rich component), which pull the injected photoelectrons out of CdSe (Se-rich component). Simultaneously, the holes prefer to transfer to CdSe (Se-rich component). Hence, the existence of type II band alignment promotes separation of electron-hole pair and enhances the free carrier density. The quantitative values of the photocarrier density and mobility are obtained from the best fitting parameters using Drude-Smith model^32^ (the solid lines in Fig. 3):


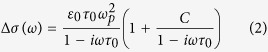


in which *C* represents the extent of carrier localization or backscattering in nanowires, *τ*_*0*_ is the scattering time and 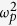 is the plasma frequency. Both the photocarrier density (
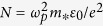
) and corresponding mobility (*μ* = *eτ*_0_(1 + *C*)/m*) are displayed as a function of delay time in Fig. 4c. Compared to composition uniform CdS_*x*_Se_*1*−*x*_ nanowires, which possess the photocarrier density in the order of 10^17^ cm^−3^ and mobility of ~100 cm^2^V^−1^S^−1^ 20, the photocarrier density is much higher and mobility is reduced in the composition graded nanowires. The high photocarrier density of composition graded CdS_*x*_Se_*1*−*x*_ nanowires underlines the large real part of the photoconductivity. The lower value of mobility in composition graded CdS_*x*_Se_*1*−*x*_ nanowires is directly related to shorter scattering time *τ*_*0*_ and large *c* parameter in Drude-Smith model. This is possibly due to the existence of interface defects and the potential fluctuation caused by severe composition disorder in graded CdS_*x*_Se_*1*−*x*_ nanowires. Both the interface defects and potential fluctuation can act as traps or scattering centers, which will localize and/or scatter free carriers. The observation of decrease in photocarrier density with delay time indicates the recombination of the free carriers while the mobility does not vary significantly with the delay time.

**Figure 5:**
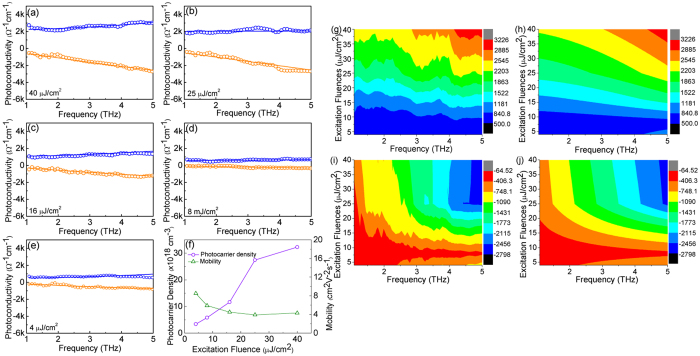
Excitation fluence dependence of the real (blue circles) and imaginary (orange circles) part of photoconductivity at a pump-probe delay time *t* = 3.5 ps. Excitation fluence from (**a**–**e**) is 40, 25, 16, 8, and 4 μJ/cm^2^, respectively. (**f**) Extracted photocarrier density (purple circles) and mobility (green triangles) as a function of the incident excitation fluence. Contour mapping of experimental ((**g**) real part, (**i**) imaginary part) and fitting ((**h**) real part, (**j**) imaginary part) results of frequency-dependent photoconductivity as a function of excitation fluences.

To further investigate the origins of the ultrahigh photoconductivity, an experiment utilizing a series of excitation fluences at the pump-probe delay *t* = 3.5 ps was carried out. The photocarrier mobility and density were derived from the least square fitting of the photoconductivity with Drude-Smith model (Fig. 5a–e). The contour mappings of the experimental and fitting results illustrate the complex photoconductivity as a function of the excitation fluences (Fig. 5g–j). Obviously, the real part of the photoconductivity with a positive value gradually increases with frequency, while the imaginary part is negative and decreases with frequency. This frequency-dependent trend is a typical feature of nanostructure photoconductivity^33^,^34^. From the best fitting parameters (Supplementary Information S3), the large negative value *C* for graded CdS_*x*_Se_*1*−*x*_ nanowires indicates that strong localization and backscattering of electrons. In addition, the value of scattering time *τ* is small and does not show significant fluctuation with the excitation fluences. Meanwhile, the plasma frequency 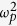 presents an increasing tendency with increasing excitation fluences. The photocarrier density *N* and mobility *μ* were extracted from the best fitting of the photoconductivity spectra and displayed as a function of excitation fluences in Fig. 5f. The photocarrier density significantly increases with excitation fluences whilst the variation of mobility shows a much more gradual decreasing trend. Therefore, the ultrahigh photoconductivity of the graded CdS_*x*_Se_*1*−*x*_ nanowires is dominated by the high photoexcited free carrier density. The relative low mobility value at higher excitation fluences is related to the shorter value of scattering time τ_*0*_ and the larger negative value of parameter *C*, indicating high degree of free carrier collision/scattering. High excitation fluence will induce more free carriers which enhance free carrier scattering/collision inside the nanowires and leads to a shorter scattering time τ_*0*_ and larger parameter *C*. Hence, the mobility shows a decrease trend with photocarrier density.

## Conclusions

In this work, we have measured the photoconductivity in bandgap-graded CdS_*x*_Se_*1*−*x*_ nanowires using OPTP spectroscopy technique. The carrier dynamics of these CdS_*x*_Se_*1*−*x*_ nanowires are clearly revealed *via* systematic analysis of the collected spectra. Ultrahigh photoconductivity as high as 2000 Ω^−1^cm^−1^ is observed in the bandgap-graded CdS_*x*_Se_*1*−*x*_ nanowires. This is attributed to the high photocarrier density caused by the formation of a series of type II band alignment along these nanowires. Our results experimentally affirm the superior photoelectrical property of bandgap-graded CdS_*x*_Se_*1*−*x*_ nanowires. This property is a promising attribute and it enhances their potentials in nanoscale optoelectronic devices such as ultrafast switches, phototransistors and solar cells. These results help to elucidate fundamental carrier dynamic processes in complex nanostructure system and would provide the theoretical support to design, fabricate and optimize optoelectronic devices using these nanomaterials.

## Additional Information

**How to cite this article**: Liu, H. *et al*. Ultrahigh photoconductivity of bandgap-graded CdS_*x*_Se_*1−x*_ nanowires probed by terahertz spectroscopy. *Sci. Rep.* **6**, 27387; doi: 10.1038/srep27387 (2016).

## Supplementary Material

Supplementary Information
