# Supplementary material for: Ultrahigh photoconductivity of bandgap-graded CdSxSe1−x nanowires probed by terahertz spectroscopy
Source: Sci Rep. 2016 Jun 6;6:27387. doi: 10.1038/srep27387 (PMC4893690; doi:10.1038/srep27387)
Supplement: Supplementary Information [file srep27387-s1.pdf]

Supplementary Information

Ultrahigh photoconductivity of bandgap-graded CdS_xSe_{1-x} nanowires probed by THz spectroscopy

Hongwei Liu,[1] Junpeng Lu,[2*] Zongyin Yang,[3] Jinghua Teng,[1] Lin Ke,[1] Xinhai Zhang,[4] Limin Tong,[3*] Chorng Haur Sow[2*]

[1]Institute of Materials Research and Engineering, Agency for Science, Technology and Research (A*STAR), 2 Fusionopolis Way, Innovis, #08-03, Singapore

[2]Department of Physics, 2 Science Drive 3, National University of Singapore, 117542, Singapore

[3]State Key Laboratory of Modern Optical Instrumentation, Department of Optical Engineering, Zhejiang University, Hangzhou, Zhejiang, 310027, China

[4]Department of Electrical and Electronic Engineering, South University of Science and Technology of China, 1088 Xueyuan Road, Nanshan District, Shenzhen, Guangdong, 518055, China

S1. Comparison of normal and bandgap-graded CdS_xSe_{1-x} nanowires.

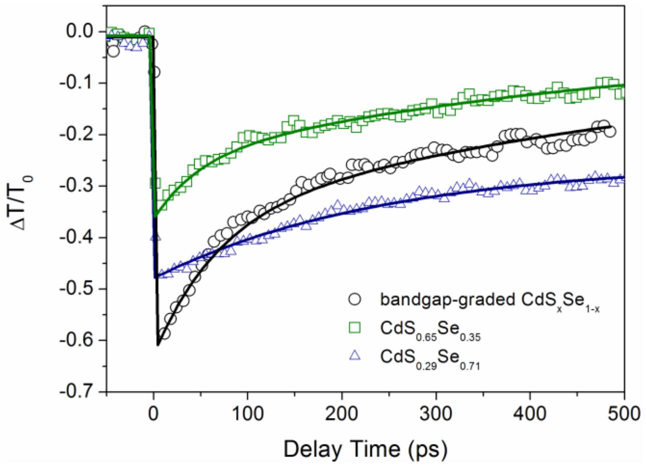

Figure S1a. Time-dependent differential THz transmission of bandgap-graded CdS_xSe_{1-x}, $CdS_{0.65}Se_{0.35}$, and $CdS_{0.29}Se_{0.71}$ nanowires at 40 μJ/cm^2. All transients are well fitted by a biexponential function.

Table S1b. Extracted parameters from the fitted biexponential function: τ_1, τ_2 and $A_1/(A_1+A_2)$.

Sample	τ_1	τ_2	$A_1/(A_1+A_2)$
Bandgap-graded CdS_xSe_{1-x}	68±5	691±20	0.40±0.06
$CdS_{0.65}Se_{0.35}$	175±26	1359±82	0.41±0.04
$CdS_{0.29}Se_{0.71}$	99±11	1042±56	0.45±0.05

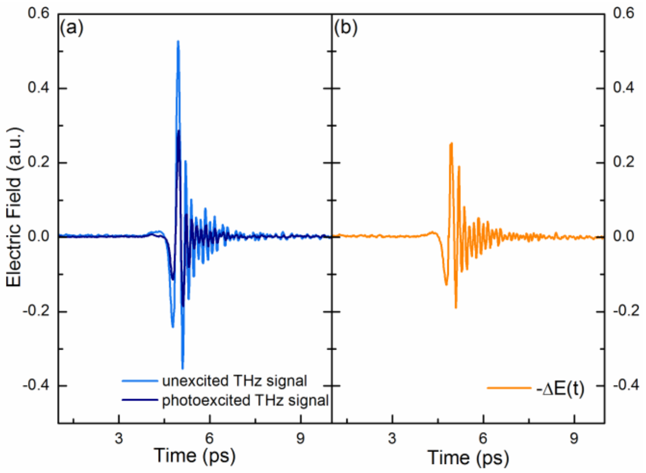

Figure S2. (a)Time-dependent THz pulse transmitted through bandgap-graded CdS_xSe_{1-x} nanowires before and after photoexcitation. (b) The transient change of THz pulses in (a).

S3. Best fitting parameters of the photoconductivity of bandgap-graded CdS_xSe_{1-x} nanowires at different incident excitation fluences using Drude-Smith model.

Incident excitation fluences (μJ/cm^2)	$\omega_p/2\pi$ (THz)	τ (*fs*)	*c*
40	113.9±17.2	4.9±2.0	-0.90±0.03
25	105.0±14.8	5.0±0.9	-0.91±0.04
16	60.7±8.4	6.9±1.4	-0.91±0.04
8	48.9±13.8	6.3±2.2	-0.84±0.07
4	37.0±7.8	8.9±1.7	-0.86±0.05
